# Licensed Practical Nurses' (LPNs') Evaluations of the Attractiveness of Work and Wellbeing at Work: A Cross-Sectional Nationwide Study

**DOI:** 10.1155/2024/3432230

**Published:** 2024-08-16

**Authors:** Mia Roos, Juho Kopra, Terhi Tevameri, Sari Viinikainen, Lauri Kuosmanen

**Affiliations:** ^1^ University of Eastern Finland (UEF) Department of Nursing Science, Kuopio, Finland; ^2^ University of Eastern Finland (UEF) School of Computing, Kuopio, Finland; ^3^ Economic Development and Employment, Espoo, Finland; ^4^ Tehy-The Union of Health and Social Care Professionals in Finland, Helsinki, Finland

## Abstract

**Background:**

Severe challenges in recruiting and retaining healthcare workers exist on a global level and are especially noticeable in elderly care services. Previous studies have not assessed how the attractiveness of a job is related to wellbeing at work among licensed practical nurses (LPNs) even though these professionals are vital in providing care to the elderly.

**Objective:**

The purpose of this study is to define factors that affect LPNs' attractiveness of work and wellbeing at work.

**Design:**

A cross-sectional survey study. *Participants*. A large-scale nationwide sample of 10 848 licensed practical nurses (LPNs) working in Finland.

**Methods:**

An online survey for LPNs working in Finland was conducted. Criteria for a good workplace (CFGW), measuring the attractiveness of work and wellbeing at work, were utilized. Descriptive and comparative analyses were performed. Cronbach's alpha values were tested to assess internal consistency (*α* = 0.739–0.915). An ANOVA or *t*-test result which indicated a statistically significant between-group difference (which was in line with difference ≥0.1) was considered as both statistically significant and significant in practice.

**Results:**

The core group, LPNs' wellbeing at work (*m* = 4.36), is extremely important factor of attractiveness of work. Regarding the subgroups high quality of care (*m* = 4.61), reconciling of work and private life (*m* = 4.59) and well-functioning practices (*m* = 4.55) were most important. Well-functioning practices associated with several background variables, e.g., with working for full-time (*p* ≤ 0.001, mean = 3.10) and age (*p* < 0.001). LPNs over 56 years old, especially, regard well-functioning practices important (*p*=3.17). LPNs' who were 56 years old or older (*m* = 3.12) and those who had work experience 1 year or less (*m* = 3.19) stated rewarding work most important.

**Conclusions:**

The core group, wellbeing, is an exceptionally important for the LPNs attractiveness of work. The LPNs' wellbeing at work is supported by the quality of care and reconciliation of work and private life.

## 1. Background

The social and healthcare industry is facing severe challenges in recruiting and retaining a skilled workforce, and this is a global phenomenon. As such, there is a high demand for healthcare professionals, qualified registered nurses (RNs) and licensed practical nurses (LPNs), with this demand only expected to increase [1, 2]. These workforce shortages particularly threaten elderly care services since LPNs and RNs are the largest professional groups in healthcare, representing over half of the global workforce [3]. The WHO estimates that there will be a global lack of 10 million qualified healthcare workers by 2030 [4]. As such, the healthcare industry needs 43 million additional healthcare workers to achieve the targeted level of healthcare [5]. It is worrying that access to quality healthcare services, which is considered a basic human right, cannot be ensured for everyone, and immediate action is needed to ameliorate this lack of access to equal healthcare [1, 3].

The global demand for qualified nurses is growing rapidly due to several reasons, including population ageing. However, shortages appear when the demand for healthcare professionals exceeds the number of available employees [3]. Every country in the world is burdened by the growing size of the elderly population. For instance, the WHO estimates that “1 in 6 people in the world will be aged 60 years or over” by 2030. According to WHO's ageing data portal, the amount of people 60 years and older will double to 2.1 billion by 2050 [6]. The greatest changes are expected for low- to middle-income countries around the world, e.g., countries in Middle Asia, South America, and West Africa [6, 7]. Healthcare systems are also burdened by challenges in replacing retiring employees and securing care for the elderly; this is clearly evident in modern welfare states such as Finland [8]. Furthermore, the COVID-19 pandemic highlighted the importance of healthcare personnel and their role in safeguarding the health of the population [9].

The impending healthcare crisis can be linked to the fact that many Organisation for Economic Co-operation and Development (OECD) member countries have not modelled the demand and supply for healthcare services up until 2025 [10]. It is also noteworthy that the definition of workforce shortage differs between countries, policies, and healthcare systems [1]. Typically, the financial and demand pressures associated with workforce shortages are characterized using criteria such as “vacancy fulfillment” or “volume of current vacant post” [11]. In addition, calculating the difference between the number of required healthcare professionals (demand) and the number of available qualified professionals (supply) is used as a policy contingent measure [1]. The occupation shortage lists published by governments also influence decision-making by, for example, the WHO and World Bank [12]. The already noted shortages in the workforce mean that countries must cooperate to create both a national system that meets the demand, as well as a resilient international network that ensures equal access to healthcare [1, 7].

The LPN role needs better recognition if healthcare systems are to overcome the nursing shortage [2]. The WHO has also noted that nursing professions, in general, require more appreciation to make the job desirable to future healthcare professionals [3]. Aiken et al. [13] concluded already in the year 2013 that without improvements in work environment nurse shortages are expected. Nurses' dissatisfaction at work is associated with the quality of care as well [14]. Addressing nursing shortages requires empirical data about how LPNs perceive their role in health care and the attractiveness of LPNs' work based on the long-term work satisfaction and motivation [15]. Moreover, promoting the wellbeing of the healthcare workforce is essential in retaining professionals in the industry. Nurses', working continuously with patients, have the best information for health care leaders in decision making to overcome critical workforce challenges motivation [13]. Current work environment and role confusion does not support wellbeing of nursing workforce to the fullest potential [15, 16].

Evidence-based research, alongside concrete actions, about the attractiveness of healthcare work is needed to retain and attract qualified professionals to the industry [12]. Hence, it is important to focus on LPNs, who have a major role in caring for the elderly population. At present, there is a clear lack of data about a vital group of healthcare professionals, the LPNs. Although the discrepancy between the demand and supply of nurses is a global phenomenon, little research has focused on the factors which promote the attractiveness of the professional and wellbeing at work [3, 17]. Understanding the factors which affect the attractiveness of a profession and wellbeing at work are essential to workforce planning [3]; to this end, Roos et al. [18] recently performed a literature review to clarify these factors. The review produced data about the factors associated with the attractiveness of social and healthcare work from LPN perspective, which correspond well with WHO's report “Health and care workforce in Europe: Time to act”; the authors of the report stressed that all European countries will face severe challenges in retaining healthcare professionals and outline 10 policies for strengthening the healthcare workforce [4, 18].

### 1.1. Theoretical Background

LPNs role as providers of care for the elderly has become increasingly important worldwide. The LPNs' work and the role as a caregiver is emphasized in Finland, as well as with the number of employees. LPNs represent the largest group of healthcare professionals in Finland, with 89 820 LPNs registered in 2021 [19]. In the same year, registered nurses (RNs) were the second largest group of employees with 76 851 RNs in total [19]. LPNs work in various fields of social and healthcare, and especially the importance of their work is visible in long-term care settings and home care [15, 19].

In this study, a definition of licensed practical nurse (LPN) is used. The LPN profession is regulated by Finnish legislation and requires a specific education (Finnish National Agency for Education). Internationally, as described by the NCSBN, licensed practice/vocational nurses' (LPN/VN) have a regulated education [20]. Globally, LPNs' education has definitions, such as enrolled nurse (Australia), licensed practical nurses (Finland, USA), licensed vocational nurses (USA), and nurse associates (UK).

The demand for LPNs has rapidly grown, especially due to population ageing. According to analysis, there is shortage of 9 000 LPNs in the Finnish public sector [21]. The social and healthcare sector employed over 415 000 people in 2022, making it Finland's largest industry [22]. By the year 2035, Finland will require over 200 000 new social and healthcare employees [23]. According to Official Statistics of Finland [22], the total number of elderly people over 75 years of age will grow rapidly, from 567 000 in 2021 to 855 000 by 2035. Finland, with a population of 5.6 million, will require a remarkably large amount of new healthcare personnel—this will require innovative strategies to ensure that the supply of workers sufficiently meets the demand.

The shortage of healthcare professionals has become a palpable problem. Already existing workforce shortages challenge nurse managers daily. Leaders in nursing are primarily responsible for the sustainable wellbeing of nursing personnel [24]. Challenges regarding the division of work with insufficient resources have a negative effect on the quality of care [15, 24]. In addition, citizens and family members have overcome worries about how general health care will be affected by these shortages [13, 17]. Strategies to resolve workforce shortages, including defining which factors promote the attractiveness of health care work, remain incomplete, yet under continuous development [1, 3, 13]. The WHO has called for more studies on the productivity of nursing professionals and the scope of practice of different professions, including LPNs, to improve the attractiveness of work [3].

Theoretical models to understand the fundamental concepts of employees' motivation, experiences, and work satisfaction have been developed at different fields of expertise. Most interestingly, the job characteristic model (JCM), the demand-control-support (DSC) model, or the theory of motivation explore and analyze the employees' experiences. The job characteristic model (JCM) implies on work characteristics (skill variety, task identity, task significance, autonomy, and feedback) and their impact on motivation, satisfaction, and performance [25]. The demand-control-support (DCS) model, instead, was developed to understand the relationship between job characteristics and employee health [26]. The theory of motivation [27] has been utilized in studies regarding healthcare workers job satisfaction, and particularly motivation and employees' attitude towards the work and work environment [28, 29]. The theory is typically applied, when investigating motivation related to work. The theory explains employees' job satisfaction/dissatisfaction to motivation. To understand the human resource experience in the context of nursing, the classic Herzberg's two-factor theory (originally developed in 1959), the theory of motivation is a well-justified framework in our study [27, 30].

There is insufficient evidence-based research into interventions for attracting and retaining healthcare workers [31]. In this study, we explore factors affecting the attractiveness of work and wellbeing at work from the LPN perspective. Furthermore, we gain insight into LPNs' work satisfaction to develop long-term work satisfaction and motivation. We explore the motivation-hygiene theory as a theoretical framework of this research to gain insight into both motivator factors and hygiene factors [27]. The theory implies that two sets of factors influence employees' motivation and work satisfaction, i.e., the motivator factors, which relate to the content of work (e.g., recognition, achievement, and responsibility) and the hygiene factors, which relate to the surrounding of work (e.g., working conditions, salary, and interpersonal relations) [27].

The theory of motivation explains that employee satisfaction, motivation, and commitment are primarily promoted by factors related to work content, such as recognition, opportunities for advancement, or responsibility [27]. Furthermore, the theory of motivation argues that income is the most important factor in attracting professionals. However, this is not the only factor, as motivational factors related to work content and a long-term career (sense of personal achievement, promotion, and stimulating work) are also highly important [28]. Furthermore, possibilities for career progression and professional development opportunities support motivation in practice [15, 18, 32]. According to a survey distributed among Swedish mental health nursing staff's salary correlates with job satisfaction [33].

A recent study found that LPNs' intention to leave the nursing profession is correlated with both emotional exhaustion and high workload [34]. Other researchers found that the limited resources of healthcare systems mean that the division of work must be harmonized better [18]. In addition, nursing education should comprehensively describe different roles, while qualified professionals who have graduated should be afforded opportunities for continuous education [17, 35]. This would maintain a clear vision of the LPN role and hopefully improve employee retention [17]. Wellbeing at work is enhanced through active collaboration with educators and employers. Work satisfaction is improved by enabling flexible career paths and educational possibilities [36]. LPNs, for instance, have more limited opportunities for career progression and LPNs lack flexible career paths when compared to RNs [15, 35].

The WHO [36] introduced a model for workforce planning in nursing which stresses comprehensive development and understanding, with national demographical data considered a means to identify the core issues of work [36]. In line with the theory of motivation, to understand the factors affecting the attractiveness of LPNs work, these interventions should first cover roles of different professionals and also education and career options [27, 36]. Weaver et al. [15] discovered that the LPNs' and employers lack a clear vision of the LPNs' role. Furthermore, the LPNs work regularly “outside” their role, despite the regulations, due to situations in patient care [15, 18]. Challenges in delivering nursing care with insufficient human resources impacts the role of an LPN, as well as and may lead not only to a role confusion but also dissatisfaction in work-related well-being [13, 15]. An Australian literature review [17] revealed enablers and barriers to the recruitment and retention of enrolled nurses (ENs or global equivalents). A clear description of the role of an LPN affects positively to the retention and recruitment of nursing professionals [15, 17]. Confusion about the scope of practice and feelings of being undervalued negatively influence the LPNs wellbeing at work and retention instead [32]. Thus, evidence of how LPNs perceive their role is essential and important in retention of professionals [37].

Comprehensive workforce planning requires deep understanding of the core purpose of work, as well as a grasp of the current psychological and practical environment [1, 3]. Hence, healthcare professionals and nurse managers should clearly understand and state the roles of different nursing professions if care activities are to be efficiently carried out [15, 27]. Although LPNs' role is expanding, the LPNs experience role confusion and thus aspire recognition as an important member of a care team [15]. Feeling empowered enhances wellbeing at work; for this reason, the role of an LPN must be clearly described in a way that does not merely repeat the legislation [15, 38, 39]. Unfortunately, not all of the possibilities of the LPN role are used in practice [15, 18]. A Finnish study [38] investigated LPNs', RNs', and managers' assessment of the activities of practical nurses. In line with the study of Weaver et al. [15], the results showed that other healthcare professionals need more information about the role of an LPN [38]. Healthcare leaders have the responsibility of clarifying and updating the scope and division of labor for the best outcome [15, 18, 38].

Nursing management and systematic workforce planning is crucial in retaining nursing professionals [40]. In addition, working conditions need to enable an adequate work-life balance since wellbeing at work deteriorates when the workload becomes a burden and/or there is not enough time to manage all of the necessary tasks [4, 13, 18]. Every healthcare organization is responsible for providing possibilities to all employees, as inefficient management narrows the possibilities to work in diverse and desired tasks [35]. This is important, as research has shown that an LPN is often considered second-level nursing position due to insufficient knowledge [18, 39]. Role clarification can maximize nursing professionals' capacity and capability; however, the scope of practice needs to be in line with organizational regulations and targets to improve the quality of patient care [15, 41]. A previous Australian study [42] attempted to improve the understanding of unclear professional roles by focusing on the scope of RNs' and enrolled nurses' (ENs) work. They found that the shortage of nursing professionals has transformed the LPN role to encompass more advanced nursing tasks [42]. More specifically, the current LPN role includes activities that were traditionally the responsibility of RNs, i.e., medication administration, care planning, and patient management [38, 42]. Clarification regarding the LPNs' expanding practical role is vital not only to improve quality of care but also for the wellbeing of the LPN [38]. This is not specific to LPNs, however, as other health care professionals also need more information about the LPN role [18]. Nursing management, which includes aspects of conflict management and dialogue with personnel, supports LPNs' organizational commitment [18, 43].

The division of healthcare professionals' work is also challenged by geographical reasons [44, 45]. The rural and remote areas of geographically vast countries, such as Finland, are characterized by insufficient access to healthcare services. Nursing managers face difficulties in recruiting and retaining nursing workforce due to geographical reasons and this will necessitate action plans to attract healthcare workers to rural areas [6, 45]. According to the WHO [6], national data on the size of the workforce are lacking. The growing demand for healthcare workers affords qualified professionals the freedom to choose countries with the most attractive salaries and workplaces and has led to rapid development in the international recruitment of health personnel [46]. A Canadian cross-sectional survey [45] examined intentions to leave among RNs, nurse practitioners (NPs), and LPNs; the results revealed that organizational commitment enhanced LPNs' intention to stay, and this finding was especially relevant for rural areas. In rural areas, workforce planning is often challenging due to geographical limitations, and the healthcare system must ensure that rural professionals have similar opportunities as urban professionals [44]. This challenges leadership to find ways in ensuring that all employees, both in rural and urban areas, feel trusted and as a long-term part of the organization [37, 45]. Feeling of being trusted enhances psychological empowerment among LPNs working in elderly care [43].

Turnover among LPNs poses a major threat to elderly care [43]. Furthermore, a shortage in nursing professionals has become evident in long-term care facilities due to the increasing number of ageing residents [2]. Havaei et al. [44] demonstrated that a good work environment reduces turnover intentions among nursing professions. Furthermore, a healthy work environment is an initial factor in supporting and enhancing LPNs' organizational commitment [44]. In promoting the human resource experience, it is essential to study the factors that affect employee motivation and commitment to work [28]. Moreover, it is important to study, namely, the experiences of caregivers regarding the attractiveness of work and factors affecting attractiveness to ensure the adequacy of healthcare workforce [13].

## 2. Purpose and Aims of the Study

The purpose of this study is to define factors that affect LPNs' attractiveness of work and wellbeing at work. This study aims to examine the factors that affect the attractiveness of work and wellbeing at work from the LPN perspective by including both demographic (e.g., age, work experience) and attractiveness (e.g., well-functioning practices and participatory management) factors in the study.

## 3. Methods

In this cross-sectional study, LPNs who are registered members either of the following two unions: the Finnish Union of Qualified Social and Health Care Professionals (Tehy) and the Finnish Union of Practical Nurses (SuPer); they were emailed a link to an online survey (*n* = 67 825). The research utilized the checklist for Reporting of Internet E-Surveys for reporting the results [47, 48] and STROBE-checklist for cross-sectional studies [49].

### 3.1. Ethical Considerations

In accordance with the ethical principles of research involving human participants and the guidelines for human sciences set forth by the Finnish National Board on Research Integrity, an ethical review statement was not required in this study since information that could be used to identify a person was not collected [50]. As the research did not require a full ethical review, the University of Eastern Finland Committee on Research Ethics provided a description of the ethical review practices in Finland. Permission to use the CFGW in this study focused on LPNs was gained from a chairperson of the Finnish Nursing Association in January 2022. Participants were given detailed information about the study prior to participation; the information document covered the aim, data collection methods, time required to complete the survey (10 min), data storage, and the researcher's (MR) contact information. Respondents were informed that submitting the survey form constitutes as an informed consent to participate in the study. Data were handled according to the EU's General Data Protection Regulation guidelines [51]. A privacy notice for scientific research was also given to respondents. In addition, all respondents were informed that participation was voluntary and responding to the survey is anonymous. Participants were not compensated for completing the survey [52, 53].

### 3.2. Sample and Sampling

Participants in this study were currently employed LPNs, who were registered members of the Union of Health and Social Care Professionals in Finland (Tehy) or the Finnish Union of Practical Nurses (SuPer). A large proportion of Finnish LPNs are registered members of the two trade unions for social and healthcare workers in Finland. A vast majority and, in our study, 90% (*n* = 67 825) of all Finnish LPNs (*n* = 79 766) employed in social and healthcare, are members either of the two trade unions [19].

To be included in this study, participants had to be licensed practical nurses (LPNs) with a vocational qualification in social and healthcare. In addition, participants had to be currently actively employed as an LPN in the social and healthcare field. Unemployed and nonworking LPNs were excluded. Other licensed health care professionals, e.g., registered nurses (RNs), were also excluded from this study. The eligibility of LPNs was filtered in advance via co-operation with the email registry holders (Tehy and SuPer). All eligible 67 825 LPNs were invited to participate online via e-mail. In total, 20 033 invitees opened the survey; 72% (*n* = 14 312) of those started filling the survey and 54% (*n* = 10 848) completed the entire survey.

### 3.3. Data Collection

Emails with a link to the online survey (in Finnish) and information about the study were sent to invitees' email addresses in the registries of the two previously described trade unions on May 10th, 2022. The online survey was accessible for two weeks (10.5.-25.5.2022). A reminder message was sent to all of the email addresses after one week. The questionnaire took about 10 minutes to complete. A total of 10 848 responses were received from LPNs and were included in the study.

### 3.4. Survey Instruments and Variables

**Figure 1:**
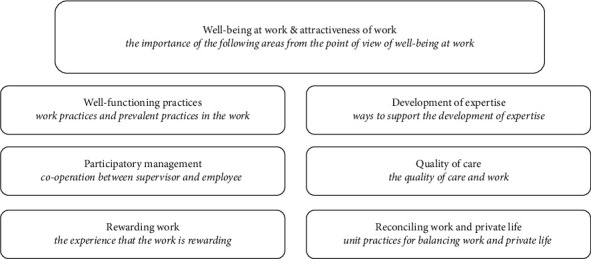
The core group and subgroups of the criteria for a good workplace (CFGW).

The survey utilized the Criteria for a Good Workplace (CFGW) instrument and questions concerning background variables. CFGW is a structured 44-item questionnaire managing wellbeing at work, with a core group (wellbeing at work) and six subgroups, which are categorized into seven theme-based subgroups. The six subgroups are well-functioning practices, participatory management, rewarding work, development of expertise, quality of care and reconciling work, and private life (Figure 1).

The core group, wellbeing at work, includes all of the subgroups as an integral part of the LPN's wellbeing at work within the CFGW. As a core group, wellbeing at work, acts as a central group for wellbeing initiatives, the subgroups. Wellbeing at work is a representative group and implies that other six groups are divisions that fall under the representation of the main group. The subgroups are specialized units with 5–9 items each to describe their specific theme. A five-point Likert scale is used to indicate the level of importance or agreement (i.e., lower values indicate lower importance or agreement, while higher values indicate higher importance or agreement).

The CFGW allows comprehensive exploration of LPNs wellbeing at work, with the core group, and the subgroups addressing specific aspects for understanding the LPN's wellbeing at work and factors enhancing attractiveness of work. (Figure 1).

The CFGW has been developed and conducted biannually by the Finnish Nursing Association for its members (RNs) since 2010. The internal validity of the CFGW has been tested, although the result of the test of validation has not been published. In our study, the CFGW was modified in 2022 and the survey aimed at LPNs for the first time. In this study, we collected an online survey data to explore LPNs' wellbeing at work. We modified the questionnaire CFGW to assess LPNs' wellbeing at work and adapted the CFGW to be suitable for LPNs with the input of a group of specialists. We pilot tested the questionnaire on vocational social and healthcare students (*n* = 13). Proofreading of the questionnaire was completed in collaboration with a vocational teacher [54].

Concerning background variables, this survey included questions about the participant's gender, age, education, employment contract (full- or part-time), type of work shift, work experience in social and healthcare and in the current unit, sector of work, number of personnel, and work unit. The background data were later split into groups for statistical analysis. Age had six groups, each with a five-year interval and ranging from 25 to 65 years. Type of work shift had the following four groups: morning shift on weekdays and weekends; two-shift work; three-shift work; and only night shifts. Work experience in social and healthcare and in the current unit had the following seven groups: 1 year or less; 2–5 years; 6–10 years; 11–15 years; 16–20 years; 21–30 years; and 31 or more years. Sector of work consisted of two groups: public and private sector. Number of personnel had the following three groups: less than 10; 10–30; and more than 30.

The data were collected, and survey was presented to invitees using Webropol [55].

### 3.5. Statistical Analysis

**Figure 2:**
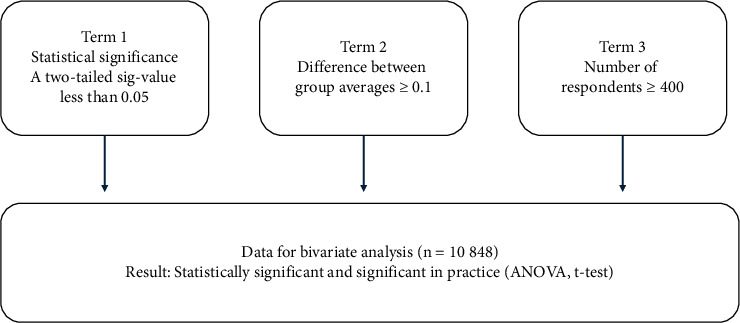
Terms of a result being statistically significant and significant in practice (sp) based on the initial power analysis.

A detailed plan for the statistical analysis was developed in collaboration with a statistician prior the starting of the study. The power analysis indicated that observing a difference of 0.1 between two-group averages would require both groups to have a sample size *n* ≥ 400 (Figure 2). Consequently, only the groups with at least 400 observations were included in the data analysis regarding *t*-test, analysis of variance (ANOVA), and CFA factor scores. Following data collection, sum variables representing the arithmetic average, were calculated for each subtopic. The background and sum variables were detailed using descriptive statistics. The internal consistency of each subgroup was assessed by calculating Cronbach's alpha.

Confirmatory factor analysis (CFA) was conducted using AMOS software (IBM, Armonk, NY). The relationships between background variables and sum variables were studied using analysis of variance (ANOVA) and *t*-tests. Due to large number of observations (*n* = 10 848), which can result in large amount of statistically significant results, we applied the concept of “significant in practice (sp)” to identify results that are not only statistically significant but also relevant to the study aims. The minimum difference regarding significance in practice (sp) in means was set at 0.1, determined after a discussion among the authors. Thus, an effect size of at least 0.125 was required for a result to be considered significant in practice if the *p* value is below threshold. An ANOVA or *t*-test result which indicated a statistically significant between-group difference (that was in line with difference ≥0.1) was considered as both statistically significant and significant in practice.

Descriptive statistics, including frequencies, percentages, mean values, and standard deviations, were used to describe the background variables. There were only four missing values out of 10 848 when calculating the sum variables, making imputation unnecessary. The normality of each sum variable was evaluated by assessing the mean value, standard deviation, skewness, and visual interpretation of histograms. All sum variables, except wellbeing at work, demonstrated a normal distribution. According to the central limit theorem, a sum of independent random variable is considered to normally distributed when the sample size is high [56, 57]. The central limit theorem's speed of convergence was evaluated using simulation for the wellbeing at work, and it was found out that 2000 observations is enough to make test statistics normally distributed.

Sum variables were calculated to study the attractiveness of work and wellbeing at work from the perspective of LPNs. Cronbach's alpha was calculated to determine whether all items grouped under a sum variable showed sufficient internal consistency. A Cronbach's alpha value over 0.70 is generally considered acceptable [58], with values in this study ranging from 0.739 to 0.915 (Table 1). No individual item had a Cronbach's alpha value exceeding that of the sum variable, so there was no reason to delete any items. One item, “the salary increases as the demands of the tasks increase” (item 24), would have resulted in a higher value for the sum variable if deleted (*α* = 0.84 vs. *α* = 0.82 if retained). Nevertheless, this item was retained in the survey since the sum variable already showed good internal consistency (*α* = 0.82).

The statistical analysis yielded seven internally consistent sum variables for items of the CFGW. The sum variables describe different aspects of the attractiveness of work and wellbeing at work in social and health care, namely, wellbeing at work, well-functioning practices, participatory management, rewarding work, development of expertise, quality of care, and reconciling work and private life (Table 1). Regarding the bivariate associations, homoscedasticity of variances was tested using Levene's test. If heterosedasticity was detected, Welch's ANOVA was applied; otherwise, traditional ANOVA was used. In these analyses, which were performed in IBM SPSS (version 27.0, IBM, Armonk, NY), the threshold for statistical significance was set as *p* ≤ 0.05.

In addition, we calculated average interitem correlation, average item-total correlation, as well as split-half reliability and composite reliability. These quantities can be found in Appendix (Supplementary file 2).

## 4. Results

### 4.1. Demographic Characteristics of Licensed Practical Nurses (LPNs)

The response rate was 16% (*n* = 10 848). Majority of the respondents were women (*n* = 10 173, 94%). The respondents' ages were divided into six groups. Over half of the LPNs were over 45 years old (54%), while a quarter were over 55 years old (25%). In contrast, a minority of respondents were under 25 years old (5%). Most of the respondents had work experience of between 6–10 (22%) or 11–15 years (19%) in social and healthcare. Moreover, 27% of the LPNs had over 20 years of work experience in social and healthcare. It was most common for the respondents to have worked at the current unit for 2–5 years (32%), while one fifth of the respondents had worked at the current unit for less than one year (Table 2).

Most of the work units had between 10–30 employees (57%), while 17% of LPNs worked in smaller work units, with less than 10 employees. The participating LPNs mostly worked in enhanced service housing for the elderly (24%) or domiciliary care (17%), and three quarters of the respondents were employed by the public sector (74%). The rest of the LPNs (26%) were working in the private sector. Of the respondents, six out of seven (86%) were educated as a qualified LPN, while the rest (14%) had completed some other vocational education (e.g., basic nurse). Most of the participating LPNs were working full-time (81%), and it was most common to have work organized over two (38%) or three shifts (37%) (Table 2).

### 4.2. Results from Bivariate Associations and Analysis of Variance

Based on the analyses, three sum variables, namely, well-functioning practices, rewarding work, and development of expertise, were most often significantly associated with independent background variables. The results revealed that the core group and dependent variable, wellbeing at work (*m* = 4.36), including its six subgroups (well-functioning practices, participatory management, rewarding work, development of expertise, quality of care, and reconciling work and private life), are extremely important for LPNs' perceptions of the attractiveness of work. Furthermore, subgroups quality of care and reconciliation of work and private life have the highest averages in wellbeing at work. (Table 1). Bivariate analyses, ANOVA, and *t*-tests were conducted to identify the variables that were independently associated with the CFGW sum scores. In this study, the presented statistically significant results are both statistically significant and meaningful in practice (mp) (Table 3 and Figure 2). Associations between the background and sum variables were assessed using bivariate analysis, analysis of variance (ANOVA), and *t*-tests. When only groups with 400 or more responses were included in the analyses, 44 of the 77 pairwise association tests demonstrated statistically significant associations that were also deemed significant in practice (Table 3 and Figure 2).

When evaluating the LPNs' experience related to rewarding work, the LPNs evaluate work rather rewarding in other areas, except the salary. Furthermore, LPNs' salary does not increase as the demands of the tasks increase (*m* = 1.64) (Table 1). Well-functioning practices need improvements according to the LPNs. Regular and joint evaluation of work processes could be improved (*m* = 2.97). Recruitment is not fully based on the competence need of the work (*m* = 2.74). Practices related to the high-quality student guidance have not been effectively agreed in the work unit (*m* = 2.98). Also, systematic activities, which support wellbeing at work, need improvement (*m* = 2.54). (Table 1). Furthermore, participatory management requires improvements in practice according to the results. LPNs strive for more open decision-making (*m* = 2.79). Furthermore, dealing effectively with problem situations requires improvements (*m* = 2.74). Consistency in following the rules needs enhancement in practice according to the results (*m* = 2.81). (Table 1). To support the LPNs' development of expertise and empowerment, systematic and sufficient orientation is important (*m* = 2.67). Furthermore, LPNs' work-related guidance is not sufficiently available for those in need (*m* = 2.93). The quality of care and work can be improved with corresponding better the number of personnel and professional structure to the demands of the work (*m* = 2.49).

All the following associations had the following *p* value: *p* < 0.001. Well-functioning practices associated with education, full-time work, type of work shift, age, work experience in social and healthcare and in the current unit, work unit, and professional pride. Rewarding work, on the other hand, was significantly associated with education, full-time work, work experience in social and healthcare and in the current unit, work unit, and professional pride. Furthermore, development of expertise was found to be significantly associated with education, full-time work, type of work shift, age, work experience in social and healthcare and in the current unit, number of personnel, work unit, and professional pride, while wellbeing at work was significantly associated with gender, age, and professional pride. (Table 3).

The independent variable full-time work demonstrated statistically significant associations with most of the sum variables (5 out of 7). LPNs who worked full-time were characterized by consistently higher averages across all the sum variables when compared to LPNs working part-time. For instance, LPNs who work full-time had significantly higher values for the sum variables of well-functioning practices (*m* = 3.1), participatory management (*m* = 3.16), rewarding work (*m* = 2.99), development of expertise (*m* = 3.18), and quality of care (*m* = 3.4) than LPNs working part-time. (Tables 1, 3). The LPNs working full-time evaluate the quality of care (*p* ≤ 0.001, *m* = 3.4) as the most important aspect of work; rewarding work (*p* ≤ 0.001) showed a statistically significant difference relative to responses from part-time workers but had the lowest average value (*m* = 2.99) (Table 3).

Age and work experience (both in social and healthcare as well as the current work unit) were associated with all the sum variables interestingly in a J-curve manner; an exception was well-being at work. Hence, people with only a few years of experience, as well as younger employees, show higher averages than people with 2–15 years of experience or between the ages of 25–55; this trend then reverses later, with the highest average values often seen for people who are nearing retirement. (Table 3).

Respondents with a vocational education other than a licensed practical nurse demonstrated systematically higher averages across all the sum variables when compared to LPNs. Among these respondents, the development of expertise was statistically significantly associated with the independent variable education (*p* ≤ 0.001) and experienced as the most important aspect (*m* = 3.28). Work unit and all the sum variables, with the exception of wellbeing at work, demonstrated statistically significant associations. Furthermore, the results indicated that the opinions of the attractiveness of work were rather similar across all units. For instance, enhanced service housing and domiciliary care, which represented most of the respondents, showed similar results as the other units. The independent variable, number of personnel, was significantly associated with rewarding work (*p* ≤ 0.001), quality of care (*p* ≤ 0.001), and reconciling work and private life (*p* ≤ 0.001). LPNs who worked in small units (less than 10 employees) demonstrated higher average values for the sum variables relative to LPNs working in larger units, which indicates higher satisfaction in smaller units (Table 3).

Interestingly, wellbeing at work showed exceptionally high averages (*m* = 4.36). This indicates that wellbeing at work is, in the opinion of LPNs, the most important aspect of attractiveness of work. (Table 1). The individual variables gender (*p* ≤ 0.001) and age (*p* ≤ 0.001) were significantly associated with wellbeing at work (Table 3). Furthermore, the subgroups' high quality of care and reconciliation of work and private life showed the highest average values (Table 3). The independent variable professional pride was significantly associated with all seven sum variables, the core- and subgroups. The LPNs who reported feeling professional pride, which was 77% of respondents, had higher average values for all seven sum variables when compared to LPNs who either reported not feeling professional pride or being unsure (Table 3).

Structural validity was evaluated using CFA, with the results presented in more detail in the Supplementary material (Supplementary file 1).

## 5. Discussion

This study has identified factors which affect the attractiveness of work and wellbeing at work from the LPN perspective. Interestingly, we found out that wellbeing of the LPNs showed exceptionally high averages. This finding supports our study aims and indicates that wellbeing at work is, in the opinion of LPNs, the most important factor of attractive work.

LPNs' empowerment and feeling of rewarding work is vital in wellbeing at work in social and healthcare. Notably, according to the results, LPNs' wellbeing at work is the most important factor of attractive work. Furthermore, the results of this study show that nurse managers' actions in systematic supporting of the LPNs' wellbeing at work need improvement in practice. The individual variable age was significantly associated with wellbeing at work. This finding indicates interestingly that young and older LPNs have similarities in regarding wellbeing at work. Furthermore, the subgroups high quality of care and reconciliation of work and private life showed the highest average values.

According to the results of this study, improvements in salaries are an important element in enhancing the feeling of rewarding work for LPNs. A recent Taiwan study showed that high salary lowers the risk of turnover, especially among newly employed nurse aids [59]. Low salary is major reason for newly licensed registered nurses for leaving the current position as well [60]. Low salary enhances LPNs' dissatisfaction at work and creates a risk, as well as for the LPNs' retention in the workplace [17]. Salary is not merely the only factor, which increases LPNs' rewarding work. Instead, the results show that nursing managers should improve actively all areas of the LPNs' wellbeing at work in practice.

This study has clarified the subareas which most impact LPNs' perceptions of the attractiveness of work. LPNs experience the work rewarding in many areas as well. The LPNs' experience, for instance, that they have the possibility to do the work well and that their work is respected. The results imply that nursing managers should take active actions to maintain the LPNs' perceived feeling of being respected and further improve all areas of rewarding work in practice. In line with Loes and Tobin [43], feeling of being trusted enhances psychological empowerment among LPNs working in elderly care. The results support the fact that nursing managers should work actively and find ways to improve LPNs' salary to improve the attractiveness of work. “Out of the box” strategies, alongside with better salaries, to improve the work environment are essential in overcoming nursing shortages [61].

The WHO has stated that national healthcare systems must have sufficient capacity and quality [3]. However, equal healthcare coverage requires a qualified workforce that meets the demand for services [5]. LPNs' perceptions of the factors that contribute to the attractiveness of the professional has not previously been studied; this current research, a large-scale nationwide study, contributes to closing this knowledge gap by including LPNs from various care settings, which is important as LPNs represent the largest group of professionals in elderly care in Finland. On an international level, the LPN role must be clarified, especially in the context of elderly care, to develop healthcare [34]. Previous studies have included a relatively small number of participants and are typically limited to RNs or specific contexts [13, 17]. A shortage of healthcare professionals is inevitable [1]. The WHO [3] outlines that major investments in healthcare systems are needed. Furthermore, it is important to consider that 90% of the nursing workforce is made up of women [3], as in our study. In addition, it is typical that nursing professionals earn salaries that are below the average wage of the country [3]. The results in this study also indicated that LPNs feel that their salaries are not in line with the tasks that they perform. According to the results, the LPNs' salary does not increase as the demands of work grow.

According to Hertzberg's two-factor theory of motivation-hygiene, salary as one of the hygiene factors influences employees' dissatisfaction at work. An employee's motivation can be enhanced by factors called motivation factors, which are related to the content of work [27]. However, the reported results are not completely in accordance with the classic theory of motivation. Previous study, which was aimed at mental health nursing personnel, found a correlation between salary and job satisfaction [35]. In this study, according to the LPNs, all the factors which positively influence personal wellbeing are extremely important in work related attractiveness. In addition, Aiken et al. stated that personnel shortages can be tackled using simple interventions, such as career advancement possibilities and evidence-based human resource management [13]. Interestingly, LPNs evaluated that all aspects of wellbeing at work (e.g., well-functioning practices, rewarding work, and participatory management) are extremely important for their personal wellbeing, which is in line with Aiken et al. [13]. Furthermore, LPNs evaluated quality of care as the most important individual factor in this study.

The findings underline that actions which promote wellbeing at work are crucial to retaining healthcare professionals. The results agree with what was reported by Aiken et al., more specifically, widespread dissatisfaction among nurses regarding patient safety and nursing care. In addition, nurses from several countries, including Finland, only judged the quality of care as poor or fair [13]. The results from this study show that LPNs prioritize patient safety and high-quality care. Also, the results indicate the nurse managers to lead more effectively high-quality student guidance and orientation processes in practice. Previous research underlines that each healthcare role, including the LPN, should have a clearly defined scope of practice if a high-quality care is to be provided [15, 42].

Based on the results, the attractiveness of work was defined based on six work-related areas, namely, well-functioning practices, participatory management, rewarding work, development of expertise, quality of care, and reconciliation of work and private life. Together, these form a structural basis for the LPNs wellbeing and can be utilized in managing the wellbeing.

Interestingly, the youngest and oldest LPNs gave similar evaluations of which factors most affect wellbeing and attractiveness of work. These respondents also gave the highest averages for current wellbeing and attractiveness of work based on the assessed subareas. Especially, young and older LPNs are a valuable resource in managing the shortfall of healthcare personnel [15, 60]. Furthermore, investments in staffing in nursing homes improve the quality of care [13, 62]. Furthermore, the responses of inexperienced and experienced LPNs showed similar trends in this study. Also, LPNs working in small units reported higher average values than peers in larger work units. The LPNs' feeling of professional pride showed a positive association with all of the wellbeing subareas. Results indicate that improving LPNs' feeling of professional pride can significantly promote the attractiveness of work and have a positive effect on the retention. In addition, leaders in nursing should improve well-functioning practices by improving the evaluation of work processes together and regularly with the LPNs.

Although this study focused on the LPN perspective, the results can be generalized to other instances in which there is a shortage of healthcare professionals. The results provide evidence that the LPNs would support any actions by nurse leaders that would improve the quality of healthcare. A recent literature review by Roos et al. showed that the work-related attraction factors do exist and can be measured [18]. The present study, which focused on LPNs' perceptions, provided unique insight into LPNs' perceptions of work-related wellbeing and the attractiveness of their profession. The findings of our study underline the importance of wellbeing of the LPNs as highly supportive element in developing the attraction of work in social and healthcare.

## 6. Strengths and Limitations

This study and its results have several strengths and limitations. One significant strength is the detailed modification of the CFGW questionnaire by a group of specialists, ensuring its suitability for LPNs. In addition, we successfully involved LPNs on a large scale, which is notable compared to previous studies [34]. The CFGW questionnaire demonstrated acceptable internal consistency (*α* = 0.739–0.915), supporting the reliability of our results [63]. In this study, we utilized the Checklist for Reporting Results of Internet E-Surveys (CHERRIES) to improve the quality of the online survey [47].

Response rates for online surveys can vary widely from 25–35% [58], while our study reached merely 16% but the completion rate and participation rates were acceptable, 54% and 72%, respectively [47]. We succeeded to invite over 90% of the target population to our study and received 10 848 in total. Thus, the data have a significant amount and portion of LPNs, which allows us to study many aspects of the phenomenon of our interest. The results of this study have potential to be generalized in Finland and potentially in other countries with similar healthcare setting. Information about the nonrespondents is not available, and this weakens the generalizability of the results [64].

We consider the response rate (16%) to be eligible for assessing relationships between different variables in these data. When interpreting, for example, means, the reader should not take them as precise as they would be if we had much higher response rate. Instead, there may be bias in the means but that does not necessarily mean that there is bias in the relationships of variables. In this study the usage of sum variables, representing average of multiple items, does reduce overall bias. For example, if one item had some bias, it will not play large role in the sum variables. Furthermore, the bias in the relationships of different variables would be present only if nonresponse would occur highly associated differently with one category of a background variable. For example, if we want to know if male and female LPNs report their wellbeing at work to be on the same level on average, it is enough that willingness to participate is same for men and women with similar wellbeing at work.

To enhance the response rate, multiple approaches were included. First, the number of questionnaire items was considered when selecting an instrument. The modified questionnaire (CFGW) includes a moderate number (44 in total) in line with recommendations [47, 65]. Second, a reminder notification was sent to respondents after one week, which can positively influence the response rate [64, 66]. Respondents were also able to review and change their answers before submitting likely enhancing the participation rate [47, 65]. Furthermore, all the survey responses were completely anonymous; one limitation regarding the anonymity is that one respondent may provide duplicate responses if a respondent uses another browser. However, in online survey, this is very unlikely [47, 55]. To ensure that duplicate answers were minimized, an IP check setting was selected prior to sending out the first emails.

This survey was distributed in May, which is the first month of the vacation period in Finland and this could negatively influence the response rate. Furthermore, the LPNs who received the survey are registered members of either of the two main trade unions for nursing personnel in Finland and pay the membership fee, which could improve participation. The survey was distributed about two years after the start of the COVID-19 pandemic, which was a strong burden for healthcare personnel and their wellbeing at work [9]. As such, it could mean that personnel would be more open to sharing their assessments of work attractiveness and wellbeing. Furthermore, the two main nurses' trade unions and the municipal and welfare area employers had strong disagreements during the spring 2022 in Finland. This resulted in nurses' trade unions giving a large strike warning in March 2022, which included up to 40 000 RNs and LPNs [67, 68]. In response, the Finnish Ministry of Social Welfare and Health ratified a law in April to ensure immediate patient safety in light of the actions of trade unions [69]. These actions most likely affected LPNs' motivation to change the healthcare system in Finland, which was in crisis, and could be expected to have positively affected the response rate. However, we do not have similar national data regarding the LPNs, which strengthens the value of our findings.

The results of this study have potential to be generalized, with some restrictions, to social and healthcare settings in Nordic countries (Finland, Sweden, and Norway). The healthcare system is decentralized, predominantly tax based and publicly provided. Furthermore, the scope of practice and the division of healthcare personnel is rather similar in the Nordic areas [70]. LPNs are key participants of the nursing workforce in USA as well. The scope of practice and role of the LPNs needs more careful assessment in many states [15, 41]. Hence, the results of this study can possibly give valuable information for a better understanding of the essential role of the LPNs and the retention of the LPNs. In online surveys, the respondents are not selected through probability sampling, which has a negative effect of the generalizability of the results [64]. The results of this study are best applied among evidence-based practice of LPNs'. Furthermore, this study is replicable, which contributes to generalizability of the results [71].

## 7. Conclusions and Future Research

There is a gap in research concerning the attractiveness of the LPNs work and the wellbeing at work of an LPN. This study helped to bridge this gap by applying the instrument CFGW to examine LPNs' perceptions of the attractiveness of their work and wellbeing at work. The research revealed several factors which affect the attractiveness of the LPN role and wellbeing at work. Several independent variables showed significant associations with the dependent variables describing attractiveness of work and wellbeing. The participating LPNs shared that personal wellbeing is exceptionally important element regarding the attractiveness of work. LPNs' wellbeing was further supported by the quality of care and reconciliation of work and private life, for instance.

This research gives important information, especially for the leaders in nursing for a better understanding about the factors promoting the attractiveness of work and wellbeing of the LPNs. This information is vital regarding, for example, the retention of the LPNs. Actions aiming to develop the work-related environment requires collaboration with the healthcare personnel, including the LPNs. Participatory management is one of the key elements to be considered. Nurse managers play a crucial role in developing the work and wellbeing of the LPNs systematically and effectively. Current environment in evidence-based practice regarding the development of LPNs' work needs better characterization. To this end, the findings of this study suggest that statistical methods can be applied to quantitatively investigate the attractiveness of nursing and wellbeing of the LPNs. Furthermore, in this study, specific factors were identified in line with the core aim of this study, which was to define factors that affect LPNs' attractiveness of work and wellbeing at work. The results of this study give more insight into development of evidence-based practice in nursing and in the LPNs' work environment. In the future, it is recommended to study the LPNs' attractiveness of work with qualitative methods to confirm the results.

## Figures and Tables

**Table 1:** Descriptive statistics of the sum variables well-functioning practices, participatory management, rewarding work, development of expertise, quality of care, and reconciling work and private life, including the calculated Cronbach's alpha value.

*n* = 10 848	m (Sd)	Cronbach's alpha (^*∗*^if item deleted)
Wellbeing at work and attractiveness of work: the importance of the following areas from the point of view of your wellbeing at work (a)	4.36 (0.64)	0.876
Well-functioning practices	4.55 (0.740)	^*∗*^0.851
Participatory management	4.29 (0.868)	^*∗*^0.858
Rewarding work	4.45 (0.845)	^*∗*^0.846
Development of expertise	4.07 (0.899)	^*∗*^0.862
Quality of care	4.61 (0.713)	^*∗*^0.851
Reconciling work and private life	4.59 (0.803)	^*∗*^0.864
Well-functioning practices: work practices and prevalent practices in the work unit (b)	3.07 (0.84)	0.846
The goals and the core purpose for my work have been defined	4.00 (0.89)	^*∗*^0.844
Work processes are regularly evaluated regularly together	2.97 (1.14)	^*∗*^0.809
Personnel recruitment is based on the competence need of the work community	2.74 (1.18)	^*∗*^0.816
The practices of high-quality student guidance have been agreed upon	2.98 (1.17)	^*∗*^0.824
Existence of systematic activities that support wellbeing at work	2.54 (1.19)	^*∗*^0.804
I give and receive feedback	3.18 (1.08)	^*∗*^0.824
Participatory management: co-operation between supervisor and employee (b)	3.13 (1.00)	0.915
My supervisor has a clear understanding of what my work is	3.57 (1.23)	^*∗*^0.907
As an employee, I am encouraged to participate in planning and decision-making	3.12 (1.22)	^*∗*^0.901
Decision-making is open	2.79 (1.20)	^*∗*^0.897
Problem situations are dealt with quickly	2.74 (1.23)	^*∗*^0.899
The supervisor supports the renewal of working methods	3.26 (1.20)	^*∗*^0.898
There are open and confidential relations between the supervisor and me (as an employee)	3.63 (1.22)	^*∗*^0.902
Consistent rules are followed in my work unit; same rules for everyone at all times	2.81 (1.32)	^*∗*^0.907
Rewarding work: the experience that the work is rewarding (b)	3.00 (0.83)	0.815
I can do my job well	3.46 (1.09)	^*∗*^0.781
I feel that my work is respected	3.09 (1.17)	^*∗*^0.758
I find work empowering (energy, dedication, and immersion in work)	3.02 (1.16)	^*∗*^0.737
My work is meaningful even though I am sometimes stressed and tired	3.59 (1.05)	^*∗*^0.763
The salary increases as the demands of the tasks increase	1.64 (0.97)	^*∗*^0.838
Development of expertise: ways and means to support the development of expertise (b)	3.14 (0.79)	0.834
Orientation is systematic and sufficient	2.67 (1.12)	^*∗*^0.812
Division of tasks is based on employee training (job title)	3.08 (1.18)	^*∗*^0.821
The division of tasks is based on the skills of the employees	3.06 (1.14)	^*∗*^0.812
I have the opportunity to develop my skills	3.35 (1.13)	^*∗*^0.808
I use the latest researched and reliable information in my work	3.48 (0.99)	^*∗*^0.818
I prepare for the development discussion in advance	3.44 (1.24)	^*∗*^0.825
Development discussions are held regularly with the employee	3.06 (1.47)	^*∗*^0.823
The most experienced employees act as mentors and transfer knowhow in the work community	3.23 (1.17)	^*∗*^0.816
I get job guidance if necessary	2.93 (1.31)	^*∗*^0.817
Quality of care: the quality of care and work (b)	3.37 (0.75)	0.773
I evaluate the patient's/client's treatment using existing quality criteria	3.59 (1.02)	^*∗*^0.754
The number of personnel and professional structure correspond to the demands of the work	2.49 (1.21)	^*∗*^0.744
The skills of practical nurses correspond to the demands of the work	3.66 (1.07)	^*∗*^0.745
I use evidence-based treatment and work methods	3.73 (0.98)	^*∗*^0.747
Treatment equipment and tools are up to date	3.40 (1.13)	^*∗*^0.730
I can work in a manner that ensures patient safety	3.34 (1.14)	^*∗*^0.717
Reconciling work and private life: unit practices for balancing work and private life (b)	3.34 (0.81)	0.739
I have enough time to do my work	2.94 (1.30)	^*∗*^0.734
When planning working hours, individual needs are considered	3.24 (1.28)	^*∗*^0.678
Transition to study, work rotation, and sabbatical leave, as well as part-time retirement, is possible	3.26 (1.15)	^*∗*^0.680
Everyone has the right to family leave and to care for a sick child or loved one	3.89 (1.05)	^*∗*^0.667
Fathers are also supported in using family leave	3.34 (0.99)	^*∗*^0.705

a: level of importance (1 = not at all important, 5 = extremely important). b: level of agreement (1 = strongly disagree, 5 = strongly agree). *n* = amount of respondents. m = mean. sd = standard deviation. Cronbach's alpha: ^*∗*^if item deleted.

**Table 2:** Characteristics of respondents (*n* = 10 848).

Background variable. *N* = 10 848	*f*	%
Gender		
Female	10 173	93.8
Male	622	5.7
Other	12	0.1
I do not want to tell	41	0.4
Age (years)		
Less than 25	517	4.8
25–35	1992	18.4
36–45	2473	22.8
46–55	2985	27.5
56–65	2866	26.4
Over 66	15	0.1
Education		
Licensed practical nurse (LPN)	9278	85.5
Basic nurse^*∗*^/or other secondary/vocational education in social and healthcare	1559	14.4
LPN with an apprenticeship training of social and healthcare working in the field	11	0.1
Working full-time (100%)		
No	2083	19.2
Yes	8765	80.8
Type of work shift		
Morning shift on weekdays and weekends	2247	22.6
Two-shift work (morning and evening shift)	4071	37.5
Three-shift work (morning, evening, and night shifts)	4025	37.1
Only nightshift	305	2.8
Work experience in social and healthcare (in years)		
1 or less	235	2.2
2–5	1787	16.5
6–10	2359	21.7
11–15	2052	18.9
16–20	1517	14.0
21–30	1565	14.4
31 or over	1333	12.3
I mainly work in the following sector		
In the public sector (municipality, city, union of municipalities, state, and personnel leasing)	8005	73.8
In the private sector (company, foundation, organization, other private employer, own company, and personnel leasing)	2843	26.2
Work experience in the current work unit (in years)		
1 or less	2145	19.8
2–5	3520	32.4
6–10	2252	20.8
11–15	1364	12.6
16–20	736	6.8
21–30	470	4.3
31 or more	361	3.3
Number of personnel in the work unit		
Less than 10	1815	16.7
10–30	6169	56.9
More than 30	2864	26.4
Work unit		
Serviced housing: elderly	838	7.7
Enhanced service housing: elderly	2559	23.6
Domiciliary care	1875	17.3
Disability services	914	8.4
Care unit of a health center	449	4.1
Early childhood education	1233	11.4
Specialized medical care	752	6.9
Mental health and substance abuse unit	420	3.9
Reception work	175	1.6
School	101	0.9
Child protection	80	0.7
Family care	8	0.1
Equipment maintenance	4	0.0
Laboratory	90	0.8
First aid	23	0.2
Reserve staff	91	0.8
Personal assistant	14	0.1
Oral healthcare	398	3.7
X-ray	7	0.1
Virtual healthcare	18	0.2
I work in several different offices	58	0.5
Other	741	6.8

*f* = frequency, ^*∗*^basic nurse: professional title for licensed practical nurses before education reform during 1993.

**Table 3:** Results from bivariate analyses and analysis of variance (ANOVA and *t* test).

Background variables		Wellbeing at work		Well-functioning practices		Participatory management		Rewarding work		Development of expertise		Quality of care		Reconciling work and private life	
	*n*	Mean	P ^*∗*^sp	Mean	P ^*∗*^sp	Mean	P ^*∗*^sp	Mean	P ^*∗*^sp	Mean	P ^*∗*^sp	Mean	P ^*∗*^sp	Mean	P ^*∗*^sp
Gender			<0.001 ^*∗*^sp		0.323		0.299		0.0651		0.964		0.342		0.046^1^
Female	10173	4.44		3.07		3.13		2.96		3.14		3.37		3.33	
Male	622	4.27		3.11		3.19		2.92		3.15		3.39		3.41	
Education			0.281^1^		<0.001^1^^*∗*^sp		0.029^1^		<0.001^1^^*∗*^sp		<0.001^1^^*∗*^sp		0.001		0.001^1^
LPN	9278	4.42		3.05		3.12		2.93		3.12		3.36		3.32	
Other^*∗∗∗*^	1559	4.44		3.20		3.19		3.10		3.28		3.43		3.41	
Working full-time			0.016		<0.001 ^*∗*^sp		<0.001 ^*∗*^sp		<0.001 ^*∗*^sp		<0.001 ^*∗*^sp		<0.001 ^*∗*^sp		0.488
No	2083	4.40		3.00		3.02		2.83		3.01		3.26		3.32	
Yes	8765	4.43		3.10		3.16		2.99		3.18		3.40		3.34	
Type of work shift			0.095		<0.001 ^*∗*^sp		<0.001^1^		<0.001		<0.001 ^*∗*^sp		<0.001		<0.001 ^*∗*^sp
Morning shift	2447	4.42		3.16		3.22		3.10		3.23		3.43		3.47	
Two shift	4071	4.44		3.05		3.13		2.93		3.11		3.33		3.25	
Three shift	4025	4.42		3.04		3.10		2.90		3.13		3.37		3.32	
Nightshift	305	4.35		3.00		2.92		3.04		3.02		3.33		3.58	
Age (years)			<0.001^1^^*∗*^sp		<0.001 ^*∗*^sp		0.029		<0.001 ^*∗*^sp		<0.001 ^*∗*^sp		<0.001 ^*∗*^sp		0.036
<25	517	4.26		2.91		3.18		2.79		3.13		3.35		3.31	
25–35	1992	4.42		2.92		3.09		2.76		3.04		3.30		3.36	
36–45	2473	4.42		3.03		3.10		2.89		3.11		3.32		3.34	
46–55	2985	4.45		3.14		3.13		3.03		3.18		3.40		3.31	
56–65	2866	4.44		3.17		3.17		3.12		3.22		3.43		3.35	
Work experience			<0.001^1^		<0.001 ^*∗*^sp		<0.001^1^^*∗*^sp		<0.001^1^^*∗*^sp		<0.001 ^*∗*^sp		<0.001 ^*∗*^sp		<0.001 ^*∗*^sp
≤1	235	4.36		3.19		3.43		3.19		3.27		3.50		3.43	
2–5	1787	4.37		2.98		3.14		2.89		3.10		3.36		3.31	
6–10	2359	4.42		2.98		3.07		2.85		3.07		3.32		3.31	
11–15	2052	4.45		3.06		3.12		2.91		3.10		3.34		3.32	
16–20	1517	4.45		3.11		3.13		2.99		3.17		3.36		3.31	
21–30	1565	4.46		3.14		3.12		3.05		3.20		3.41		3.36	
31 or over	1333	4.45		3.23		3.21		3.13		3.30		3.43		3.42	
Work experience, current unit			0.037^1^		<0.001^1^^*∗*^sp		<0.001 ^*∗*^sp		<0.001^1^^*∗*^sp		<0.001^1^^*∗*^sp		<0.001 ^*∗*^sp		<0.001 ^*∗*^sp
≤1	2145	4.39		3.11		3.31		3.07		3.17		3.41		3.41	
2–5	3520	4.42		3.03		3.11		2.91		3.11		3.36		3.33	
6–10	2252	4.44		3.02		3.04		2.86		3.09		3.31		3.29	
11–15	1364	4.45		3.08		3.05		2.93		3.15		3.34		3.29	
16–20	736	4.46		3.08		3.08		3.03		3.21		3.38		3.34	
21–30	470	4.43		3.22		3.17		3.13		3.29		3.48		3.36	
31 or more	361	4.45		3.28		3.2		3.19		3.35		3.52		3.40	
Sector of work			0.694		0.500		0.028		0.189		0.001		0.994		<0.001
Public sector^*∗∗*^	8005	4.43		3.07		3.12		2.95		3.16		3.37		3.32	
Private sector^*∗∗*^	2843	4.43		3.08		3.17		2.98		3.1		3.37		3.38	
Number of personnel in the work unit			0.169^1^		0.841^1^		0.021		<0.001^1^^*∗*^sp		0.034^1^		<0.001^1^^*∗*^sp		0.002^1^^*∗*^sp
Less than 10	1815	4.4		3.07		3.15		3.06		3.12		3.44		3.37	
10–30	6169	4.43		3.07		3.15		2.94		3.16		3.36		3.35	
More than 30	2864	4.44		3.06		3.09		2.93		3.12		3.34		3.29	
Work unit			0.130^1^		<0.001^1^^*∗*^sp		<0.001^1^^*∗*^sp		<0.001 ^*∗*^sp		<0.001^1^^*∗*^sp		<0.001^1^^*∗*^sp		<0.001 ^*∗*^sp
Serviced housing	838	4.41		3.01		3.06		2.87		3.04		3.29		3.24	
Enhanced service housing	2559	4.44		2.97		3.01		2.82		3.03		3.29		3.26	
Domiciliary care	1875	4.44		2.95		3.03		2.91		3.01		3.25		3.1	
Disability services	914	4.4		3.2		3.28		3.07		3.23		3.51		3.56	
Care unit of a health center	449	4.4		3		3.05		2.81		3.12		3.27		3.24	
Early childhood education	1233	4.4		3.18		3.35		3.02		3.28		3.34		3.44	
Specialized medical care	752	4.43		3.22		3.21		3.08		3.36		3.51		3.42	
Mental health and substance abuse unit	420	4.46		3.2		3.24		3.06		3.27		3.45		3.53	
Reception work	175			3.1		3.08		3.19		3.21		3.53		3.49	
Oral healthcare	398	4.43		3.14		3.1		3.12		3.28		3.72		3.41	
Other	741	4.5		3.18		3.21		3.12		3.21		3.48		3.48	
Do you feel professional pride as an LPN			<0.001^1^^*∗*^sp		<0.001^1^^*∗*^sp		<0.001^1^^*∗*^sp		0.000^1^^*∗*^sp		0.001^1^^*∗*^sp		0.001^1^		<0.001 ^*∗*^sp
No	2398	4.39		2.70		2.74		2.37		2.77		3.02		3.06	
I cannot say	2307	4.36		2.96		3.02		2.79		3.04		3.27		3.25	
Yes	6143	4.47		3.26		3.32		3.25		3.33		3.54		3.48	

^1^ = ANOVA with Welch's ANOVA. ^*∗*^sp = significant in practice. (i) A two-tailed *p* value less than 0.05. (ii) Limit value 0.1 (difference between mean values ≤ 0.1 + *n* >v400). ^*∗∗*^Public sector: municipality, city, union of municipalities, state, and personnel leasing. ^*∗∗*^Private sector: company, foundation, organization, other private employer, own company, and personnel leasing. ^*∗∗∗*^Basic nurse: professional title for licensed practical nurses before education reform in 1993.

## Data Availability

The survey data of this study are not publicly available due to confidentiality reasons. In case of further inquiries regarding the availability of data, please contact the corresponding author.

## Additional Points

*What Is Already Known*. (i) Shortages in the healthcare workforce represent a global threat that has particularly grave implications for elderly care services, as LPNs and RNs are the largest professional groups. (ii) There is a lack of data about a vital group of healthcare professionals, the LPNs. (iii) Although the discrepancy between the demand and supply of nurses is a global phenomenon, little research has focused on the factors which promote the attractiveness of the professional and wellbeing at work. *What This Paper Adds*. (i) This study has identified factors which affect the attractiveness of work and wellbeing at work from the LPNs' perspective. (ii) The attractiveness of work was defined based on six work-related areas, namely, well-functioning practices, participatory management, rewarding work, development of expertise, quality of care, and reconciliation of work and private life. Together, these form a structural basis for an LPN's wellbeing. (iii) Statistical methods were applied to assess wellbeing among LPNs, and the results can be used to improve wellbeing in practice in addressing nursing shortages.

## Supplementary Materials

Supplementary Materials Supplementary file 1: confirmatory factor analysis (CFA) assessing the model fit. Supplementary file 2: average interitem correlation, corrected item-total correlation, split-half reliability adjusted using the Spearman–Brown prophecy formula, and composite reliability. (a) Average interitem correlation and corrected item-total correlation. (b) Split-half reliability adjusted using the Spearman–Brown prophecy formula and composite reliability.
