# Supplementary material for: Licensed Practical Nurses' (LPNs') Evaluations of the Attractiveness of Work and Wellbeing at Work: A Cross-Sectional Nationwide Study
Source: J Nurs Manag. 2024 Aug 16;2024:3432230. doi: 10.1155/2024/3432230 (PMC11918497; doi:10.1155/2024/3432230)

**Description:**

Structural validity of the survey instrument was evaluated using Confirmatory Factor Analysis (CFA). Confirmatory Factor Analysis (CFA) was conducted using AMOS software (IBM, Armonk, NY).

Interpretation of CFA is introduced by following indices of fit Normed chi-square, Comparative fit index, Tucker-Lewis’s index, root mean square error of approximation and standardized root mean square residual (See table 1 for recommeded levels and estimates). Illustration of path diagram for the analysis was conducted using IBM® SPSS® Amos (Figure 1).

Reference: Kline, R. B. 2023. Principles and practice of structural equation modeling. Fift Edition. Guilford publications.

**Supplementary file 1.** Description of confirmatory factor analysis (CFA) assessing the model fit.

**Table 1.** Key model fit indices for the model.

| **Fit indices** | **Recommended level** | **Estimate** |
| --- | --- | --- |
| Normed chi-square (Chi-square / DF) | < 5.0 | 29.793 |
| Comparative fit index (CFI) | > 0.90 | 0.889 |
| Tucker-Lewis’s index (TLI) | > 0.90 | 0.881 |
| Root mean square error of approximation (RMSEA) | < 0.08 | 0.052 |
| Standardized root mean square residual (SRMR) | < 0.08 | 0.042 |

**Figure 1.** Illustration of path diagram for the analysis conducted using IBM® SPSS® Amos.


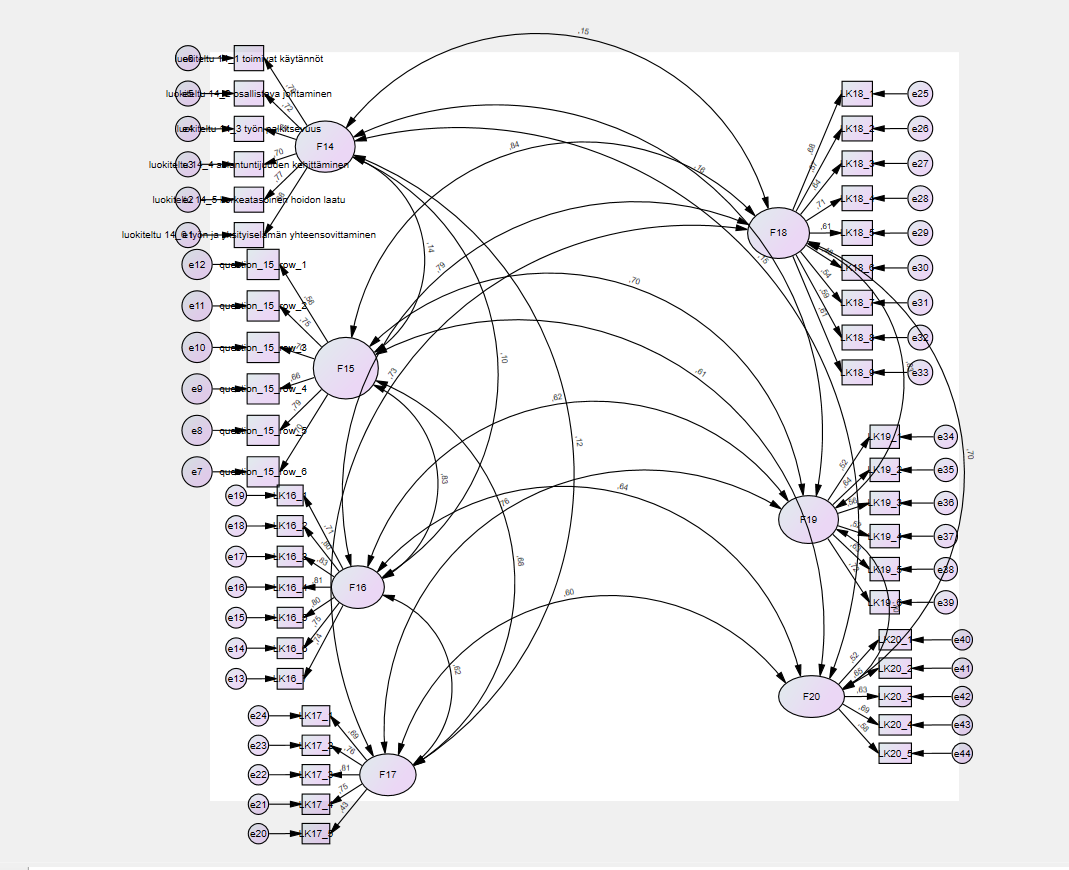

Supplement: Supplementary Materials — Supplementary file 1: confirmatory factor analysis (CFA) assessing the model fit. Supplementary file 2: average interitem correlation, corrected item-total correlation, split-half reliability adjusted using the Spearman–Brown prophecy formula, and composite reliability. (a) Average interitem correlation and corrected item-total correlation. (b) Split-half reliability adjusted using the Spearman–Brown prophecy formula and composite reliability. [file 3432230.f1.zip › Supplementary file 1. Description of confirmatory factor analysis (CFA) assessing the model fit.docx]
