# Supplementary material for: Licensed Practical Nurses' (LPNs') Evaluations of the Attractiveness of Work and Wellbeing at Work: A Cross-Sectional Nationwide Study
Source: J Nurs Manag. 2024 Aug 16;2024:3432230. doi: 10.1155/2024/3432230 (PMC11918497; doi:10.1155/2024/3432230)
Supplement: Supplementary Materials — Supplementary file 1: confirmatory factor analysis (CFA) assessing the model fit. Supplementary file 2: average interitem correlation, corrected item-total correlation, split-half reliability adjusted using the Spearman–Brown prophecy formula, and composite reliability. (a) Average interitem correlation and corrected item-total correlation. (b) Split-half reliability adjusted using the Spearman–Brown prophecy formula and composite reliability. [file 3432230.f1.zip › Supplementary file 2..docx]

Supplementary file 2. Average Inter-item correlation, Corrected Item-Total correlation, Split-half Reliability adjusted using the Spearman-Brown prophecy formula, Composite reliability

Supplementary documents:

a. Average Inter-item correlation, Corrected Item-Total correlation,

b. Split-half Reliability adjusted using the Spearman-Brown prophecy formula and Composite reliability.

Supplementary document a. Average Inter-item correlation, Corrected Item-Total correlation.

| Item | Average Inter-Item correlation | Corrected Item-Total correlation |
| --- | --- | --- |
| 14 | 0.548 |  |
| 14/1 |  | 0.713 |
| 14/2 |  | 0.668 |
| 14/3 |  | 0.734 |
| 14/4 |  | 0.650 |
| 14/5 |  | 0.715 |
| 14/6 |  | 0.628 |
| 15 | 0.475 |  |
| 15/1 |  | 0.494 |
| 15/2 |  | 0.687 |
| 15/3 |  | 0.651 |
| 15/4 |  | 0.613 |
| 15/5 |  | 0.708 |
| 15/6 |  | 0.611 |
| 16 | 0.606 |  |
| 16/1 |  | 0.686 |
| 16/2 |  | 0.746 |
| 16/3 |  | 0.785 |
| 16/4 |  | 0.763 |
| 16/5 |  | 0.771 |
| 16/6 |  | 0.732 |
| 16/7 |  | 0.695 |
| 17 | 0.461 |  |
| 17/1 |  | 0.597 |
| 17/2 |  | 0.670 |
| 17/3 |  | 0.732 |
| 17/4 |  | 0.658 |
| 17/5 |  | 0.373 |
| 18 | 0.366 |  |
| 18/1 |  | 0.588 |
| 18/2 |  | 0.508 |
| 18/3 |  | 0.585 |
| 18/4 |  | 0.627 |
| 18/5 |  | 0.540 |
| 18/6 |  | 0.470 |
| 18/7 |  | 0.509 |
| 18/8 |  | 0.550 |
| 18/9 |  | 0.544 |
| 19 | 0.363 |  |
| 19/1 |  | 0.460 |
| 19/2 |  | 0.508 |
| 19/3 |  | 0.497 |
| 19/4 |  | 0.490 |
| 19/5 |  | 0.555 |
| 19/6 |  | 0.604 |
| 20 | 0.369 |  |
| 20/1 |  | 0.407 |
| 20/2 |  | 0.541 |
| 20/3 |  | 0.535 |
| 20/4 |  | 0.581 |
| 20/5 |  | 0.471 |

Supplementary document b. Split-half Reliability adjusted using the Spearman-Brown prophecy formula and Composite reliability.

| Variable Name | Number of items included | Spearman-Brown Coefficient | Composite reliability |
| --- | --- | --- | --- |
| 14 | 6 | 0.876 | 0.879 |
| 15 | 6 | 0.833 | 0.847 |
| 16 | 7 | 0.908 | 0.915 |
| 17 | 5 | 0.805 | 0.823 |
| 18 | 9 | 0.769 | 0.838 |
| 19 | 6 | 0.770 | 0.773 |
| 20 | 5 | 0.696 | 0.751 |
